# Functional Status of Hypothalamic–Pituitary–Thyroid and Hypothalamic–Pituitary–Adrenal Axes in Hospitalized Schizophrenics in Shanghai

**DOI:** 10.3389/fpsyt.2020.00065

**Published:** 2020-02-27

**Authors:** Yuncheng Zhu, Haifeng Ji, Lily Tao, Qing Cai, Fang Wang, Weidong Ji, Guohai Li, Yiru Fang

**Affiliations:** ^1^ Clinical Research Center & Division of Mood Disorders, Shanghai Mental Health Center, Shanghai Jiao Tong University School of Medicine, Shanghai, China; ^2^ Division of Psychiatry, Shanghai Changning Mental Health Center, Affiliated Greenland Hospital of Bio-X Institute, Shanghai Jiao Tong University, Shanghai, China; ^3^ Key Laboratory of Brain Functional Genomics (MOE & STCSM), Shanghai Changning-ECNU Mental Health Center, Institute of Cognitive Neuroscience, School of Psychology and Cognitive Science, East China Normal University, Shanghai, China; ^4^ Division of Psychiatry, Shanghai Yangpu Mental Health Center, Shanghai, China; ^5^ Zhenjiang Mental Health Center, Zhenjiang, China; ^6^ CAS Center for Excellence in Brain Science and Intelligence Technology, Shanghai, China; ^7^ Shanghai Key Laboratory of Psychotic Disorders, Shanghai, China

**Keywords:** schizophrenia, thyroid function tests, pituitary–adrenal function tests, neuroendocrine system, chronic stress

## Abstract

**Objective:**

Neuroendocrine dysfunction is related to the pathogenesis of mental disorders, but conclusions from clinical research lack consistency. We aimed to investigate the neuroendocrinal pathophysiology and its correlation with clinical symptoms in patients with schizophrenia.

**Methods:**

The present cross-sectional study included 486 inpatients with schizophrenia admitted at a psychiatric hospital in Shanghai within one year, and 154 healthy controls (HC) matched on age and gender. The serum hemoconcentrations of thyroid-stimulating hormone (TSH), total triiodothyronine (TT3), total thyroxine (TT4), free triiodothyronine (FT3), free thyroxine (FT4), adrenocorticotrophic hormone (ACTH), and cortisol (COR) were measured *via* electrochemical luminescence immunoassay. Pathophysiological conversions of neuroendocrine were then associated with gender, age, age at onset, antipsychotic treatment using hierarchical multiple linear regression.

**Results:**

When compared to HC, the schizophrenia group showed elevated ACTH and COR levels and decreased TT3 and TT4 levels (*p*‘s < 0.05). First-episode patients showed lower TSH and higher FT3 and FT4 (*p*’s < 0.05) compared to recurrent patients. Female patients showed higher TSH and lower TT3, FT3, and ACTH levels (*p*’s < 0.05) compared to males. We observed the area under the curve (AUC) of the predictive model to distinguish between schizophrenia and HC to be 0.737 among total samples and between first-episode and recurrent schizophrenia to be 0.890 among subgroups.

**Conclusions:**

Decreased TT3 and TT4 and elevated ACTH and COR levels appear to be associated with schizophrenia symptoms. The chronic recurrent trait of schizophrenia may cause long-term effects on FT3 and FT4 while changes in thyroid, and adrenal function as a result of mental disorder varied with gender. The pathophysiological parameters provide fair to good accuracy of these models.

## Background

Neuroendocrine dysfunction is related to the pathogenesis of mental disease. To date, however, research findings lack evidence of clinical consistency. For example, many psychoneuroendocrinological studies have focused on treatment-naïve patients (1). Because antipsychotics need to be maintained for as long as is necessary, follow-up psychoneuroendocrinological changes are short of longitudinal evidence.

Hypothalamic–pituitary–thyroid axis (HPTA) function is significantly associated with the onset of schizophrenia, by affecting emotion regulation and cognitive functioning (2). The hypothalamic–pituitary–adrenal gland axis (HPAA) also plays an important role in the relationship between psychological stress and neural activity (3).

The neuroendocrine functions of HPTA and HPAA are involved in the regulation of moods, emotions, and cognitive behaviors in acute or chronic stress. Lowering pituitary volume in schizophrenia is most likely by enhancing stress regulation and lowering the distress due to psychotic symptoms (4). For example, clinically significant fluctuations in thyroid hormones were found after surgery for Cushing’s syndrome (5). These findings suggest that there may be multiaxial changes in pituitary function associated with the appearance of psychiatric symptoms. Most neuroendocrine studies of schizophrenia focused on antipsychotics, course of disease, symptoms, and other influencing factors as research objectives. Thyroid and adrenal function in hospitalized patients with schizophrenia may be associated with factors of disease, acute or hospitalized phase, gender, and recurrence. Women are more prone to endocrine dyscrasia. Many, however, underestimate gender differences, which affect the generalizability of results (6–8). It is important to include these variables in analyses involving schizophrenia since they are likely to be involved in the pathogenesis of psychosis.

Aside from clear-cut neuroendocrine disorders of the pituitary axes (*e.g.*, hyperthyroidism), which are shown to be high risk factors of psychiatric symptoms (9), subclinical neuroendocrinal hyper- or hypofunctions are also commonly seen in the course of diagnosis and treatment of schizophrenia (10, 11). Due to the side effects of antipsychotic drugs, cardiac deceases, diabetes or smoking, neuroendocrine diseases have a higher comorbidity in patients with schizophrenia (12), particularly disorders in HPTA and HPAA, which are closely related to the occurrence and development of schizophrenia (13, 14).

Patients with schizophrenia may self-regulate their hormone levels in the HPTA and HPAA under chronic stress exposure (15). For example, free triiodothyronine (FT3) level is positively associated with cognitive function (16), while cortisol (COR) responses to stress (17). In terms of thyroid hormonal components, conjugated thyroxine is the storage and transportation form of the hormone, while FT4 is the active component. Total thyroxine (TT4) is composed of T4 and free thyroxine (FT4), with only 0.02% of the circulating hormone being FT4 (18). T4 undergoes extra-thyroidal conversion to T3, which is three to four times more active than T4 (19). Hence, compared to T3, T4 is a more moderate adjustment for overall thyroid function. The main adrenal function, as we know, activates to boost metabolism and to increase excitability of the central and peripheral nervous systems (20). Moreover, type II iodothyronine 5′ deiodinases catalyzes the conversion of T4 to T3 (21), and chronic physiological COR increase may result in a decline in T3 by inhibiting this enzyme in schizophrenia (22).

Many studies have found sex differences in HPAA in patients with early psychosis or schizophrenia. Patients with recent onset of psychosis were observed to have a significant sex difference, with a blunted COR response to awakening in men but not in women (23), while a significantly lower dehydroepiandrosterone sulfate (DHEA-S) was found in male patients (24). COR-to-DHEA-S (C/D) ratio might predict health levels of patients (25). In the domain of executive function, COR predicted poor performance on the cognitive functioning in male schizophrenics (26). On the other hand, Labad J et al. (27) suggest that there are sex differences in the relationship between HPAA measures and cognitive abilities in early psychosis. So, there are different interpretations of HPAA by gender due to first episode or recurrent schizophrenia. Furthermore, we have noticed that the most common diseases of the neuroendocrine systems present with obvious gender differences (28) (**Table 1**).

**Table 1:** Gender ratios in common hypothalamic–pituitary–glandular axial disorders.

Disease	Gender ratio (Male : Female)
Hyperthyroidism	1:4–6
Hypothyroidism	1:4
Cushing disease	1:3
Addison’s disease	1:2–3

In short, we noticed that there was not too much on the topic and that few studies had included both first episode and chronic schizophrenics focusing on both of hemoconcentrations of HPTA and HPAA hormones. We collected them among patients with schizophrenia and healthy controls (HC). There have been numerous studies conducted over the past years that have examined HPAA and HPTA in samples of patients with schizophrenia and psychosis. Findings from these studies have been examined in meta-analyses and systematic reviews (29–31). However, many of these markers have not been examined in the same samples. We excluded potential endocrine diseases related with these two axes before analysis, and we hypothesized that schizophrenia with different episodes of disease or gender difference may have an effect on neuroendocrine function.

## Participants and Method

### Participants

The study was approved by the Institutional Ethical Committee for clinical research of Shanghai Changning Mental Health Center, Shanghai, China. Informed consent was provided according to the *Declaration of Helsinki*. We recruited 486 patients with schizophrenia and 154 healthy individuals matched on age, gender, and ethnicity (Han).

Patients were recruited from Shanghai Changning Mental Health Center, an in-patient psychiatric ward, from 01 March 2017 to 28 February 2018. There were approximately 590,000 permanent residents in the Changning district of Shanghai (32). Therefore, we expected that the psychiatric demographics was representative of that of the city of Shanghai.

The diagnosis of schizophrenia without comorbid diagnosis was ascertained according to the DSM-IV criteria. The mental examination was conducted by three-level ward round including at least one chief physician. Patient inclusion criteria were: Chinese Han ethnicity, aged 18 to 65 years, at stable phase (no more acutely symptomatic reoccurrence at the time of investigation under more than a year of stable medication adherence) or first-episode patients. Exclusion criteria were: More than one antipsychotic medication for the acute psychosis treatment in a year, first-diagnosed but not antipsychotic naïve (use of levothyroxine, antithyroid, glucocorticoid, bromocriptine, testosterone, estrogen, progestin, oral contraceptive, amiodarone, lithium, psychoactive substance or other medicines related to neuroendocrine diseases within a year); use of stimulants or inhibitors (microsomal drug metabolizing enzyme inducers or inhibitors, diuretics, or any affecting thyroid or adrenal function shown in medicine instruction); pregnant or in *postpartum* period; history of central nervous system disease; history of thyroid, adrenal, or gonad diseases tested using B-ultrasonography or immunoserology; any neuroendocrine diseases confirmed by the neuroendocrine test; Pittsburgh sleep quality index (PSQI) (33) scores >7. We defined that the first-episode patients were not on antipsychotic medication, while the recurrent patients took only one medication for at least one year.

**Figure 1:**
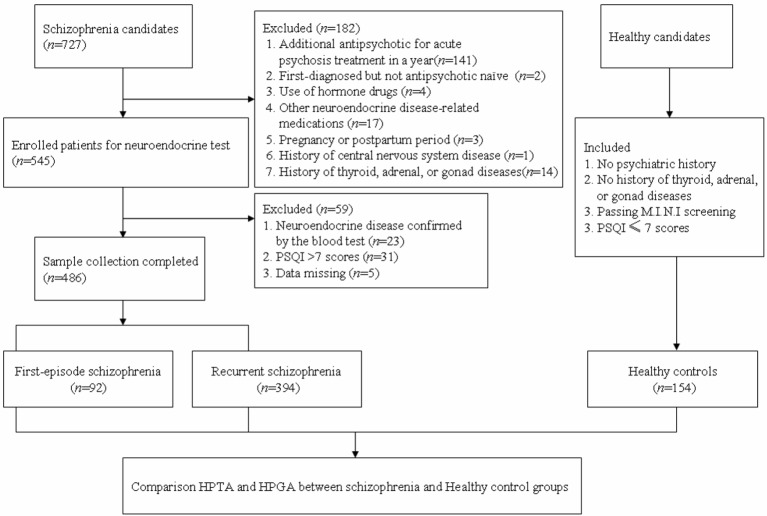
Flowchart of screening process and data classification.

Blood samples of the HC group were collected from medical examination items of the general population in Shanghai, recorded in the medical examination center of Tongren Hospital, affiliated to Shanghai Jiaotong University School of Medicine. The healthy individuals were voluntarily recruited by advertisement to participate in the study with no psychiatric history, which were excluded by a self-made questionnaire. The Mini-International Neuropsychiatric Interview (M.I.N.I) (34) and the PSQI were then used for screening any psychotic disorder of the HCs (see **Figure 1** for a flow diagram of sample selection).

### Measures

#### Positive and Negative Syndrome Scale

The Chinese Mandarin version of the Positive and Negative Syndrome Scale (PANSS) (35) has been shown to be a reliable and valid instrument for the assessment of the severity of psychopathology in hospitalized patients with schizophrenia. The scale consists of 30 items, each rated using a 7-point scale. We recorded patients’ total PANSS, positive symptoms, negative symptoms, and general psychopathology scores as variables.

#### Hemoconcentration of Hormones in HPAA and HPTA

The hormones tested include serum concentrations of thyroid-stimulating hormone (TSH), TT3, FT3, TT4, FT4, adrenocorticotrophic hormone (ACTH), and COR. We collected venous blood of the patients who must be under inpatient sleep management with good sleep rhythm, as well as healthy individuals, between 6:00 a.m. and 8:00 a.m. Blood samples were taken before breakfast to minimize the effects of circadian variation. A total of 5 ml blood was collected by a single venipuncture into yellow plain tubes (with coagulants and separation gel). After standing at room temperature for 30 min, the blood sample was centrifuged for 15 min at 1,800 g. The serum was carefully aliquoted into 2-ml screw-top microtubes for subsequent storage. Two aliquots were collected from each study individual and one for standby application. Each microtube was labeled with a coded identification label and stored at −80°C (36). The panel of 640 sera was used to measure hormone concentrations over two widely available commercial automated analyzer systems with standard procedure: Roche Cobas e601 and Modular e170 automatic electrochemiluminescence immunoassay system (ECLIA) for TSH, T3, T4, and COR and ACTH, respectively (37, 38). The hormonal assay was performed in the Lanwei Clinical Testing Laboratory, Shanghai, China. We used the reference intervals of these hormone concentrations for comparison according to the People’s Republic of health industry standards: TSH 95% CI (range, 0.27–4.20 mIU/L), TT3 (range, 1.3–3.1 nmol/L), FT3 (range, 2.8–7.1 pmol/L), TT4 (range, 66–181 nmol/L), FT4 (range, 12–22 pmol/L), ACTH (range, 7.2–63.3 ng/L) and COR (range, 171~536 nmol/L).

### Statistical Analysis

Given the relatively large sample size (640 data points for HPTA and HPAA), sample size calculation was omitted. All statistical computations were performed using SPSS 17.0. Data were represented as mean ( ± SD). Comparisons of the candidate values between patients with schizophrenia and HCs were performed *via* independent sample Student’s *t-*test or one-way ANOVA for normally distributed data, and Wilcoxon *W*-Test or Mann-Whitney *U*-test for skewed distribution data, as appropriate. Analysis of covariance (ANCOVA) was conducted for comparison of hormones in these two axes (age, BMI, drug dose as covariables), as appropriate. Chlorpromazine (CPZ)-equivalent dose was conversed for analyzing risk of antipsychotic treatment in recurrent schizophrenics (39). The differences between groups were analyzed by *post hoc* with Bonferroni correction (40). A hierarchical multiple linear regression analysis for each PANSS subscore was conducted by including HTA axis hormones (TSH, FT4, and FT3) and HPA axis hormones (ACTH, COR) and main covariates (gender, age, age at onset, and CPZ-equivalent dose). All statistical analyses were defined as two-tailed *p* value, significance level of 5% (*α* = 0.05). After normal transformation where necessary, the nonnormal distribution data were conducted with statistical disposal for *Cohen’s d* or *η* ^2^. Effect sizes were provided by OR, *Cohen’s d* or *η* ^2^.

## Results

### Comparison Between Schizophrenia and HC Groups

**Table 2** presents the statistics for the participant groups. The median of illness duration and age at onset was 9 years and 26 years old, respectively. There was no difference in age between the two groups (*p* > 0.05). Age and BMI were considered as covariables to measure the difference in these two axes between groups, and there were no group × age or group × BMI interactions (*p*’s > 0.05). The schizophrenia group showed significantly higher BMI, FT4, ACTH, and COR levels and significantly lower TT3 and TT4 levels compared to HC (*p*’s < 0.05).

**Table 2:** Quantitative comparison between schizophrenia and HC groups in the gender subgroup.

	Schizophrenics (*n* = 486, male/female = 292/194)	Healthy controls (*n* = 154, male/female = 93/61)	*Z/t/F*	*P*	*Cohen’s d*
Age (y)	39.3 ± 12.6	37.3 ± 8.1	0.687	0.492	0.064
Male	39.0 ± 12.9	38.4 ± 7.2	0.660	0.510	0.079
Female	39.8 ± 12.2	35.7 ± 9.3	1.819	0.069	0.267
BMI (kg/m^2^)	23.6 ± 4.0	22.3 ± 3.2	4.169	<0.001	0.386
Male	23.9 ± 4.0	22.7 ± 3.5	2.594	0.010	0.309
Female	23.2 ± 3.9	21.8 ± 2.6	3.416	0.001	0.501
TSH (mIU/L)	2,09 ± 1.48	1.86 ± 0.86	0.387	0.699	0.036
Male	1.85 ± 1.37	1.88 ± 0.81	1.870	0.061	0.223
Female	2.46 ± 1.58	1.83 ± 0.93	2.874	0.004	0.422
TT3 (nmol/L)	1.58 ± 0.30	1.88 ± 0.51	75.332	<0.001	0.637
Male	1.64 ± 0.30	1.92 ± 0.55	37.091	<0.001	0.560
Female	1.49 ± 0.27	1.81 ± 0.43	50.181	<0.001	0.802
FT3 (pmol/L)	4.48 ± 0.79	4.43 ± 0.97	0.914	0.339	0.062
Male	4.66 ± 0.78	4.38 ± 0.98	7.968	0.005	0.297
Female	4.21 ± 0.74	4.49 ± 0.94	7.351	0.007	0.309
TT4 (nmol/L)	103.2 ± 21.2	121.2 ± 20,2	9.148	<0.001	0.846
Male	102.2 ± 20.8	122.5 ± 20.8	7.693	<0.001	0.916
Female	104.9 ± 21.9	120.2 ± 19.5	5.024	<0.001	0.738
FT4 (pmol/L)	18.1 ± 3.4	17.3 ± 3.0	2.850	0.004	0.264
Male	18.2 ± 3.4	17.5 ± 3.0	1.812	0.070	0.216
Female	18.0 ± 3.5	17.0 ± 3.1	2.345	0.019	0.344
ACTH (ng/L)	34.3 ± 23.4	23.1 ± 9.3	5.418	<0.001	0.501
Male	39.4 ± 25.6	23.7 ± 11.3	6.213	<0.001	0.740
Female	26.8 ± 17.0	22.2 ± 4.81	0.707	0.480	0.104
COR (nmol/L)	490.2 ± 214.3	397.1 ± 75.7	38.984	<0.001	0.912
Male	500.6 ± 199.0	373.4 ± 84.3	32.637	<0.001	1.041
Female	474.7 ± 235.1	382.8 ± 60.3	3.726	<0.001	0.727

Although there were no significant differences between the two groups in TSH or FT3 levels (*p*’s > 0.05), higher TSH and lower FT3 levels of female patients were observed compared to female HC (*p*’s < 0.05), while higher FT3 of male patients was observed compared to male HC (*p*’s < 0.05). The female schizophrenia group showed higher FT4, and the male schizophrenia group showed higher ACTH compared to each isosexual HC (*p*’s < 0.05).

Mean ( ± SD) for the normal distribution data and median (Q1, Q3) for the skewed distribution data; BMI, body mass index; TSH, thyroid-stimulating hormone; TT3, total triiodothyronine; FT3, free triiodothyronine; TT4, total thyroxine; FT4, free thyroxine; ACTH, adrenocorticotrophic hormone and COR, cortisol.

We constructed a hierarchical multivariable prediction model using the TSH, FT3, FT4, ACTH, COR, gender (woman = 0, man = 1), and age for diagnosis of schizophrenia. The receiver operating characteristic (ROC) curve showed the fair accuracy of this model, yielding an area under the curve (AUC) of 0.737 (95% CI, 0.699–0.774), and the best cut-off value (Youden index) was 0.463.

**Table 3** presents the number of participants with abnormal endocrine concentrations between groups for HPTA and HPAA hormone levels outside of the upper or lower limit of the normal range. The only significant differences were TSH, TT3, FT4, ACTH, and COR levels between female groups, and TT3 and COR between male groups (*p*’s < 0.05). However, there was no difference in FT4 or ACTH level between the male subgroup as well as FT4 between female’s (*p*’s > 0.05).

**Table 3:** Qualitative comparison between schizophrenia and HC groups outside of upper or lower limit of the normal range in the gender subgroups.

	Abnormal value (*n*%)		*χ2* value	*p*	*OR*	*95% CI for OR*	
	Schizophrenics (*n* = 486)	Healthy controls (*n* = 154)				Lower	Upper
TSH	45(9.3%)	3(1.9%)	9.011	0.003	5.128	1.572	16.67
Male	19(6.5%)	3(3.2%)	1.409	0.235	2.075	0.604	7.246
Female	26(13.4%)	0(0%)	/	0.001^a^	/	/	/
TT3	95(19.5%)	7(4.5%)	19.645	<0.001	5.102	2.315	11.24
Male	44(15.1%)	6(6.5%)	4.635	0.031	2.551	1.059	6.250
Female	51(26.3%)	1(1.6%)	17.369	<0.001	21.277	2.890	166.7
FT3	6(1.2%)	6(3.9%)	3.172	0.075	0.308	0.098	0.970
Male	2(0.7%)	3(3.2%)	/	0.093^a^	0.205	0.034	1.258
Female	4(2.1%)	3(4.9%)	0.550	0.458	0.407	0.089	1.873
TT4	6(1.2%)	0(0%)	/	0.344^a^	/	/	/
Male	2(0.7%)	0(0%)	/	1.000^a^	/	/	/
Female	4(5.1%)	0(0%)	/	0.575	/	/	/
FT4	74(15.2%)	13(8.4%)	4.583	0.032	1.949	1.048	3.323
Male	38(13.0%)	7(7.5%)	2.057	0.151	1.880	0.792	4.274
Female	36(18.6%)	6(9.8%)	2.565	0.109	2.088	0.835	5.236
ACTH	70(14.4%)	6(3.9%)	12.337	<0.001	4.149	1.767	9.709
Male	38(13.0%)	6(6.5%)	3.001	0.083	2.151	0.887	5.319
Female	32(16.5%)	0(0%)	/	<0.001^a^	/	/	/
COR	236(48.6%)	6(3.9%)	99.208	<0.001	23.256	10.101	52.63
Male	138(47.3%)	6(6.5%)	50.171	<0.001	12.987`	5.495	30.30
Female	98(50.5%)	0(0%)	/	<0.001^a^	/	/	/

a p was calculated by Fisher’s exact test.

### Comparison Between First-Episode and Recurrent Schizophrenia Subgroups and HCs

The median ages of the recurrent schizophrenia group and the HC group were 15 and 13 years older, respectively, than that of the first-episode schizophrenia group (*p* < 0.001). The BMI showed no change between the first-episode schizophrenia and the recurrent schizophrenia subgroups (*p =* 0.001). The median CPZ-equivalent dose of the recurrent schizophrenia group was 328.3 (mg/day). Total PANSS score and its sub-scales showed no differences (*p*’s > 0.05).

Age and BMI were considered as covariables to measure the difference in these two axes between groups, and there were no group × age or group × BMI interactions (*p*’s > 0.05). The subgroups of schizophrenia showed significantly higher ACTH and COR levels and significantly lower TT3 and TT4 levels compared to HC (*p*'s < 0.05), but there was no difference in those levels between subgroups of schizophrenia (*p*’s > 0.05). The first-episode and HC groups showed significantly lower TSH level than that in the recurrent group (*p* < 0.05) while the first-episode group showed significantly higher FT3 and FT4 levels compared to recurrent schizophrenia and HC (*p*’s < 0.05). See **Table 4**.

**Table 4:** Comparison between first-episode and recurrent schizophrenia subgroups and HCs.

	Schizophrenics		Healthy controls (n = 154)	*Z/t/F*	*p*	*Corhen’s d/*η^2^	*Post Hoc^a^*
	First-episode (n = 92)	Recurrent (n = 394)					
Age (y)	27.2 ± 7.0	42.1 ± 12.0	37.3 ± 8.1	156.670	<0.001	0.330	1 < 3 < 2
BMI (kg/m^2^)	23.6 ± 4.1	23.6 ± 4.0	22.3 ± 3.2	6.924	0.001	0.021	1,2 < 3
Total PANSS	85.7 ± 9.8	85.5 ± 11.8	/	0.153	0.878	0.018	/
PANSS positive	19.2 ± 5.2	19.6 ± 7.0	/	0.445	0.656	0.052	/
PANSS negative	17.5 ± 5.2	17.8 ± 5.1	/	0.357	0.721	0.041	/
PANSS general	37.7 ± 4.7	37.6 ± 5.4	/	0.222	0.825	0.026	/
TSH (mIU/L)	1.68 ± 0.95	2.19 ± 1.57	1.86 ± 0.86	5.826	0.054	0.021	1,3 < 2
TT3 (nmol/L)	1.61 ± 0.31	1.57 ± 0.29	1.88 ± 0.51	38.016	<0.001	0.112	1,2 < 3
FT3 (pmol/L)	4.67 ± 0.85	4.44 ± 0.78	4.43 ± 0.97	3.239	0.040	0.010	2,3 < 1
TT4 (nmol/L)	106.7 ± 20.7	102.4 ± 21.3	121.6 ± 20.2	85.993	<0.001	0.127	1,2 < 3
FT4 (pmol/L)	19.4 ± 3.7	17.8 ± 3.3	17.3 ± 3.0	16.943	<0.001	0.036	2,3 < 1
ACTH (ng/L)	33.2 ± 21.9	34.6 ± 23.7	23.1 ± 9.3	29.536	<0.001	0.051	3 < 1,2
COR (nmol/L)	512.7 ± 173.7	485.0 ± 222.6	397.1 ± 75.7	21.083	<0.001	0.063	3 < 1,2

a Bonferroni correction for multiple comparisons was applied, and the result was p < 0.05; BMI, body mass index; PANSS, Positive and Negative Syndrome Scale; TSH, thyroid-stimulating hormone; TT3, total triiodothyronine; FT3, free triiodothyronine; TT4, total thyroxine; FT4, free thyroxine; ACTH, adrenocorticotrophic hormone and COR, cortisol.

We constructed a hierarchical multivariable prediction model using the TSH, FT3, FT4, ACTH, COR, gender, and age as diagnostic predictor of first-episode and recurrent schizophrenia. The ROC curve shows the good accuracy of this model, yielding an AUC of 0.890 (95% CI, 0.855–0.924), and the best cut-off value (Youden index) was 0.700.

### Comparison Between Genders in the Schizophrenia Group

Of the 486 patients, 292 cases (60%) were males. There was no difference in demographic characteristics between the female and male patients with schizophrenia, including age, illness duration, first-episode age, BMI, and CPZ-equivalent dose (*p*’s > 0.05). Age, BMI, and CPZ-equivalent dose were chosen as covariables to measure the difference in these two axes between groups. There were sex × age and sex × BMI interactions (*p*’s < 0.05), but not sex × CPZ-equivalent dose interaction (*p* > 0.05). Positive symptom score and TSH level in the female schizophrenia group were significantly higher (*p*’s < 0.05), while negative symptom score, TT3, FT3, and ACTH levels were significantly lower than those in the male schizophrenia group (*p*’s < 0.05). See **Table 5**.

**Table 5:** Comparison between genders in the schizophrenia group.

	Schizophrenics		*Z/t*	*P*	*Corhen’s d*
	Female (n = 194)	Male (n = 292)			
Age (y)	39.8 ± 12.1	39.0 ± 12.9	0.532	0.595	–
Course (y)	13.2 ± 12.7	11.0 ± 10.6	1.743	0.081	–
Age at onset (y)	26.6 ± 8.2	28.0 ± 8.5	1.881	0.060	–
Body Mass Index (kg/m^2^)	23.2 ± 3.9	23.9 ± 4.0	1.752	0.080	–
CPZ-equivalent dose (mg/day)^a^	325.0 ± 143.9	332.0 ± 152.0	0.463	0.644	–
Total PANSS	85.2 ± 11.3	85.8 ± 11.6	0.590	0.555	0.055
Positive symptoms	20.5 ± 5.5	18.9 ± 7.3	2.603	0.010	0.241
Negative symptoms	16.7 ± 4.6	18.4 ± 5.4	3.775	0.000	0.350
General psychopathology	37.2 ± 5.1	37.9 ± 5.3	1.555	0.121	0.144
TSH (mIU/L)	2.45 ± 1.58	1.85 ± 1.37	5.113	<0.001	0.488
TT3 (nmol/L)	1.49 ± 0.27	1.64 ± 0.30	11.394	<0.001	0.503
FT3 (pmol/L)	4.21 ± 0.74	4.66 ± 0.78	13.474	<0.001	0.587
TT4 (nmol/L)	104.8 ± 21.9	102.2 ± 20.8	1.503	0.133	0.132
FT4 (pmol/L)	18.0 ± 3.5	18.2 ± 3.4	0.584	0.559	0.083
ACTH (ng/L)	26.8 ± 17.0	39.4 ± 25.6	5.994	<0.001	0.588
COR (nmol/L)	474.7 ± 235.1	500.6 ± 199.0	1.758	0.079	0.132

PANSS, Positive and Negative Syndrome Scale; TSH, thyroid-stimulating hormone; TT3, total triiodothyronine; FT3, free triiodothyronine; TT4, total thyroxine; FT4, free thyroxine; ACTH, adrenocorticotrophic hormone and COR, cortisol; CPZ, chlorpromazine; a, derived from the antipsychotic treatment in the recurrent schizophrenia subgroup (*n* = 394, male/female = 222/172).

### Correlations Between Clinical Features and HPTA and HPAA Hormone Levels in the Schizophrenia Group

The hierarchical multiple linear regression models of the three regression analyses (PANSS positive, PANSS negative, PANSS general) are shown in **Table 6**. These equations were constructed by *X_1_* = TSH, *X_2_* = FT3, *X_3_* = FT4, *X_4_* = ACTH, *X_5_* = COR, *X_6_* = gender, *X_7_* = age, *X_8_* = age at onset, *X_9_* =BMI, *X_10_* = CPZ-equivalent dose (*p*’s < 0.05). The regression equations were finally observed as follows:

$$\text{Logit}(\text{Positive})=10.987-0.031X_{1}+0.075X_{2}+0.200X_{3}+0.003X_{4}+0.003X_{5}-1.620X_{6}+0.077X_{7}-0.014X_{8}-0.097X_{9}-0.003X_{10};$$

$$\mathrm{Logit}(\text{Negative})=20.639-0.061X_{1}-1.472X_{2}+0.143X_{3}+0.014X_{4}+0.001X_{5}+2.224X_{6}+0.004X_{7}-0.033X_{8}-0.027X_{9}+0.002X_{10};$$

$$\text{Logit}(\text{General})=35.158-0.132X_{1}+0.051X_{2}-0.015X_{3}-0.004X_{4}+0.004X_{5}+0.711X_{6}+0.037X_{7}-0.008X_{8}-0.025X_{9}+0.001X_{10}.$$

**Table 6:** Hierarchical multiple linear regression analysis of HPTA and HPAA hormone levels for each PANSS subscore.

Model	*β^a^*	S.E.	*β^b^*	*t*	*p*
PANSS positive					
Constant	10.987	3.211		3.422	0.001
TSH	−0.031	0.214	−0.007	−0.143	0.886
FT3	0.075	0.418	0.009	0.178	0.858
FT4	0.200	0.096	0.102	2.087	0.037
ACTH	0.003	0.015	0.010	0.195	0.845
Cortisol	0.003	0.002	0.082	1.625	0.105
Gender	−1.620	0.687	−0.118	−2.358	0.019
Age	0.077	0.029	0.148	2.671	0.008
Age at onset	−0.014	0.042	−0.018	−0.333	0.739
BMI	−0.097	0.082	0.055	1.191	0.234
CPZ-equivalent dose	−0.003	0.002	−0.077	−1.599	0.110
PANSS negative					
Constant	20.639	2.425		8.509	<0.001
TSH	−0.061	0.162	−0.018	−0.375	0.708
FT3	−1.472	0.316	−0.227	−4.660	<0.001
FT4	0.143	0.072	0.095	1.979	0.048
ACTH	0.014	0.011	0.066	1.308	0.192
Cortisol	0.001	0.001	0.027	0.553	0.580
Gender	2.224	0.519	0.212	4.285	<0.001
Age	0.004	0.022	0.009	0.172	0.863
Age at onset	−0.033	0.032	−0.054	−1.047	0.296
BMI	−0.027	0.062	−0.020	−0.432	0.666
CPZ-equivalent dose	0.002	0.001	0.059	1.248	0.213
PANSS general					
Constant	35.158	2.543		13.824	<0.001
TSH	−0.132	0.170	−0.037	−0.776	0.438
FT3	0.051	0.331	0.008	0.154	0.878
FT4	−0.015	0.076	−0.010	−0.193	0.847
ACTH	−0.004	0.012	−0.017	−0.322	0.768
Cortisol	0.004	0.001	0.166	3.267	0.001
Gender	0.711	0.544	0.066	1.307	0.192
Age	0.037	0.023	0.090	1.607	0.109
Age at onset	−0.008	0.033	−0.013	−0.238	0.812
BMI	−0.025	0.065	−0.018	−0.383	0.702
CPZ-equivalent dose	0.001	0.001	−0.027	−0.563	0.574

^a^Unstandardized Coefficients. ^b^Standardardized Coefficients.

TSH, thyroid-stimulating hormone; TT3, total triiodothyronine; FT3, free triiodothyronine; TT4, total thyroxine; FT4, free thyroxine; ACTH, adrenocorticotrophic hormone and COR, cortisol; PANSS, Positive and Negative Syndrome Scale.

In the subscales, the score of positive symptoms was positively correlated with FT4 level, woman, and age (*p*’s < 0.05). The negative symptom score was negatively correlated with FT3 level (*p* < 0.05) and positively correlated with FT4 level and man (*p* < 0.05). The general psychopathology score was positively correlated with COR levels (*p* < 0.05). The *r* values of the three models were 0.239, 0.290, and 0.194, respectively.

## Discussion

At first, we analyzed the overall level of these hormones in **Table 2** and the probability of abnormal value in **Table 3** between schizophrenia and HC groups. Age and BMI were considered as confounders, so we tried to eliminate them using the ANCOVA for normally distributed data. While the differences were statistically significant, the clinical significance of these differences remains questionable since the mean values were within the normal laboratory reference range. Therefore, it might help to compare if the rates of abnormal values were greater/lesser in the schizophrenia group using the laboratory cut-off values. Results showed that qualitative values of TSH were affected by gender, whereas the other converted qualitative values for HPTA hormones were consistent with their quantitative data. The Fisher’s exact test was applied because of the small expected sample size of abnormal TT4 values. For HPAA, ACTH qualitative data of female patients differed from the corresponding quantitative data, whereas for male patients, they were consistent. This may be due to the skewed distribution of ACTH, in which the most elevated hormone levels were still within the normal range (41). The other converted qualitative data were consistent with quantitative results.

The elevated FT4 level among schizophrenics compared to HC is not enough to overcome the overall reduction in TT4 levels. The increased FT4 may be overconsumed by humoral regulation for meeting the needs of pathological and physiological conditions. Thus, TT4 is drained continuously through the active transformation. The chronic toxicological change of the active component FT4 may be one of the causes of chronic psychopathology in schizophrenia (42). The present pattern of TT4 reduction was observed among patients with schizophrenia with a median illness duration of nine years. A longitudinal study had previously confirmed increased hypothyroidism as the disease progresses (43). It is also possible that hypoalbuminemia, hypotransthyretin (44) and a variety of drugs (androgens, glucocorticoids, growth hormones, and so on) can reduce the thyroglobulin content (45), producing a false-positive or false-negative outcome in HPTA among patients with schizophrenia. Transportation is the main factor to explain the difference between serum thyroid function and actual thyroid hormone state of brain tissue. Transthyretin synthesized in the choroid plexus is involved in movement of thyroxine from the blood into the cerebrospinal fluid and the distribution of thyroid hormones in the brain. Besides the serum concentration of thyroid hormones, the dysregulation of transthyretin plays several roles in neurobiological function of schizophrenia, including neurodevelopment and endocrine disruption (46).

Classic negative feedback regulation cannot explain the synchronous increasing levels of ACTH and COR in the present study as they are negatively correlated. It may be that the synthesis, storage, reabsorption, decomposition, and release process of thyroid hormone depend on humoral regulation for a long period of time (19). Furthermore, illness duration can lead to long-term changes that reform endocrine negative feedback. For acute onset, therefore, negative feedback regulation shows little effect on the secretion of TSH at neuroregulation stage (47).

Under stress, both ACTH and COR levels increased in schizophrenia by means of depending on neural regulation (48), which is more rapid than humoral regulation. The peripheral hypersecretion of both FT4 and COR can maintain excitability of the nervous system. The thyroid–adrenergic interactions produce heat, maintain body temperature, and coordinate emotional regulation (49). The elevated COR level in the acute phase is in line with traditional biomedical models in the developmental course of schizophrenia (50). However, the adrenal fascicular cells present a pulse synthesis of COR, so the secretion is not only regulated by ACTH, but also regulated by vasopressin secreted by hypothalamus (51), stress (52), expression of peripheral clock genes (53), and so on. There is a diurnal variation, a COR awakening response. The secretion of COR appears to be aligned with circadian rhythm fluctuations and seasonal differences. After controlling the time for collecting samples, this result may explain the coelevation of ACTH and COR levels with both acute and chronic stress.

In terms of disease progression, FT3 and FT4 levels of acute stage patients were higher than those of chronic patients, consistent with previous findings of elevated levels of thyroxine during the acute stage of the disease (42). These findings suggest that the chronic course of the disease may have long-lasting effects on HPTA. In the present study, the chronic schizophrenic course presented a median illness duration of nine years. However, the free components of triiodothyronine and thyroxine reduced to normal levels and deactivate the biological changes from negative feedback and also have an effect on inhibiting metabolism, reducing the excitability of central and peripheral nervous systems. On the other hand, Vedal et al. (54) found that lower FT4 is associated with use of antipsychotics, which is contrary to our results in general. Out first-episode schizophrenic patients had never taken antipsychotics, and elevated FT4 is responsible for the hypermetabolism at the acute stage in particular. The effect of FT4 was then clarified after comparison among the first-episode schizophrenia, recurrent schizophrenia, and HC groups. In **Table 2**, the standardized mean effect sizes (*Cohen’s d)* of TT3, TT4, FT4, ACTH, and COR range from 0.264 to 0.912 of different clinical relevance. This was also the case for gender subgroups when comparing between schizophrenia and HC. However, subgroup analysis in **Table 3** indicates that the effect size is miniscule. This suggests that while the differences are statistically significant, they are likely of weak clinical relevance in different periods of schizophrenia. Therefore, we assume that the gender factor may play a more important role.

The main analyses also explored differences between diagnostic groups with a sex-stratified approach. From another perspective of morbidity, thyroid-related diseases in women have an incidence 5–20 times more than in men (55). At certain physiological periods such as pregnancy, HPTA function becomes altered. The increase of estrogen during this period can result in increases in thyroxine-binding globulin (56) and risk of schizophrenia (57). Recent studies have established models in pregnant and nonpregnant women, suggesting that pregnancy factors could result in decreases in FT4 level (58). We excluded pregnant women for homogenous of samples. Then, we tested potential interactions with sex (age, BMI, and CPZ-equivalent dose) and found that sex difference is interacted with age and BMI in analyzing TT3 and FT3, but not with medication. Results from subgroup analysis also showed gender differences in HPAA and HPTA (24). We alleviated part of the drug effect by means of conversing CPZ-equivalent dose between gender subgroups. Then, compared to male patients, TSH level of female patients was higher, while TT3, FT3, and ACTH levels were lower. *Cohen’s d* values of TSH (0.488), TT3 (0.503), FT3 (0.587), and ACTH (0.588) showed relatively clear differences when comparing between genders in the schizophrenia group in **Table 5**. Principles of the autoimmune system indicate immune changes for females after puberty and in the *postpartum* period by increasing 5-HT (59). The elevated ACTH and T3 (high functioning) may play important roles in the acute illness of male hospitalized schizophrenics with an intense stress response (1). The reference ranges of those axes should, therefore, be set out for schizophrenia separately for the genders. Some have suggested that there is an alteration in levels of HPAA hormones based on the menstrual cycle (60). Further, a study of chronic stress and the HPAA found that individuals who had suffered child maltreatment also presented with gender differences in HPAA function; namely, COR activation responses in females were significantly slower than in males (61). In the aspect of molecular structure, sex hormones and glucocorticoids both belong to neurosteroids. For example, mifepristone achieves its antiprogesterone effect by competing to bind to progesterone receptor while it also shows the ability of attaching to glucocorticoid receptors (62, 63). Affected by the concentration of these hormones, nonspecific receptors may produce different biological effects, which can better explain the higher morbidity of neuroendocrine diseases in women. Due to the special complexity with large physiological fluctuations of hormone levels in the menstrual cycle, the functional study of sex hormones was very limited in schizophrenia research.

We observed the AUC of the predictive model to distinguish between schizophrenia and HC to be 0.737 among total samples and between first-episode and recurrent schizophrenia to be 0.890 among subgroups. The pathophysiological parameters provide fair to good accuracy of these models, with great improvement in the subgroups. A study indicated an association between use of antipsychotics and lower FT4 (54). Although we are unable to clarify the risk of psychotropic pharmacy, the changes of HPTA and HPAA have taken place. Age, gender, TSH, FT3, FT4, ACTH, and COR took effect on the discrimination models.

From the perspective of clinical medicine, the severity of schizophrenia symptoms may be correlated with the clinical features of hospitalized patients and their hormone levels in HPTA and HPAA (42). Looking at PANSS subscales, the weak correlations between positive symptom score and FT4 level and gender, between negative symptom score and FT3, FT4 levels and gender, and between general psychopathology score and COR level remained. It indicates that thyroid hormones may be associated with main symptoms of schizophrenia. Severity of symptom is associated with increased FT4 level in male schizophrenia (64), while thyroid dysfunction (TSH, FT3, and FT4) is associated with schizophrenia, especially in female patients (65).

The general psychopathology score reflects the emotional response and part of the cognitive functioning of patients, with higher scores previously found among those who attempt suicide (66). The present findings indicate that the chronic stress reaction of schizophrenia maintained at a high level for an extended period of time, and is directly related to the severity of psychotic symptoms. Elevated COR levels during the disease likely impair cognitive functions by producing chronic stress.

## Conclusion

The present study compared hormone levels in HPTA and HPAA between patients with schizophrenia and HCs, between female and male patients, and between first-episode and recurrent patients. We also examined relationships between patient clinical features (age, illness duration, PANSS scores, and subscale scores) and HPTA and HPAA hormone levels. Decreased TT3 and TT4 levels and elevated ACTH and COR levels were found to be associated with the hospitalized patients. In addition, in this specified research on schizophrenia, both thyroid function and adrenal function varied with gender to some extent. Further studies are needed to fully explain the differences observed. The recurrent and chronic trait of schizophrenia may cause long-term effects on thyroid function, such as impacting TSH, FT3, and FT4 levels. Lastly, we found that symptom severity evaluated by the PANSS in hospitalized patients yielded a weak linear correlation with COR level. Looking at PANSS subscales, the weak correlation with COR level remained for the general psychopathology score.

### Limitations

The use of antipsychotic drugs in patients with mental disorders affects their neuroendocrine function (54). Therefore, we recruited first-episode schizophrenic patients who had never taken antipsychotics or recurrent schizophrenic patients who had used medication in a lower dose for at least one year because they had experienced the acute phase and the consolidation phase. Samples chosen from the consolidation phase can avoid excessive interference of multiple antipsychotics on the axes. Hence, the endocrine effect of drugs was not studied in this study in order to avoid confounding caused by analysis of different drugs between groups. Transformed variables using CPZ-equivalent dose (39) may reduce the impact of confounder, but cannot eliminate it. We hope to include patients in different phases of the illness in future studies. If resources allow, we recommend a longitudinal study design involving patients who are in a stable phase initially and treated with only one antipsychotic medication.

## Data Availability Statement

All datasets generated for this study are included in the article/supplementary material.

## Ethics Statement

The studies involving human participants were reviewed and approved by the Institutional Ethical Committee for Clinical Research of Shanghai Changning Mental Health Center, Shanghai, China. Written informed consent to participate in this study was provided by the participants' legal guardian/next of kin.

## Author Contributions

YZ and FW designed the study, collected and analyzed data, and wrote the first draft of the manuscript. HJ contributed to data collection and statistical analysis. LT, QC, WJ, and GL discussed and commented on the manuscript. YF reviewed and edited the manuscript. All authors read and approved the manuscript.

## Funding

The work was supported by the National Key Research and Development Program of China (2016YFC1307100), the National Natural Science Foundation of China (81771465, 81930033), the National Key Technologies R&D Program of China (2012BAI01B04), the Sanming Project of Medicine in Shenzheng (SZSM201612006), the Innovative Research Team of High-level Local Universities in Shanghai, the Innovation Team Project of Shanghai Changning District, and the Project of Shanghai Yangpu Mental Health Center (YJY2018-3).

## Conflict of Interest

The authors declare that the research was conducted in the absence of any commercial or financial relationships that could be construed as a potential conflict of interest.

## Abbreviations

HPTA, hypothalamic–pituitary–thyroid axis; HPAA, hypothalamic–pituitary–adrenal gland axis; HPGA, hypothalamic–pituitary–gonad axis; COR, cortisol; HC, healthy controls; PANSS, Positive and Negative Syndrome Scale; TSH, thyroid-stimulating hormone; TT3, total triiodothyronine; FT3, free triiodothyronine; TT4, total thyroxine; FT4, thyroid hormone; ACTH, adrenocorticotrophic hormone.
